# Lumpy Skin Disease Virus Infection Activates Autophagy and Endoplasmic Reticulum Stress-Related Cell Apoptosis in Primary Bovine Embryonic Fibroblast Cells

**DOI:** 10.3390/microorganisms11081883

**Published:** 2023-07-26

**Authors:** Jinlong Tan, Yinju Liu, Weike Li, Yongzhi Zhang, Guohua Chen, Yongxiang Fang, Xiaobing He, Zhizhong Jing

**Affiliations:** State Key Laboratory for Animal Disease Control and Prevention, Key Laboratory of Veterinary Public Health of Agriculture Ministry Lanzhou Veterinary Research Institute, Chinese Academy of Agricultural Sciences, Lanzhou 730046, China; tjlong14@163.com (J.T.);

**Keywords:** lumpy skin disease virus, autophagy, endoplasmic reticulum, apoptosis

## Abstract

Poxviruses have been associated with humans for centuries. From smallpox to mpox to lumpy skin disease virus (LSDV), members of the poxvirus family have continued to threaten the lives of humans and domestic animals. A complete understanding of poxvirus-mediated cellular processes will aid in the response to challenges from the viruses. In this study, we demonstrate that LSDV infection results in an abnormal ultrastructure of the endoplasmic reticulum (ER) lumen in primary bovine embryonic fibroblast (BEF) cells, and we further show that an ER imbalance occurs in LSDV-infected BEF cells. Additionally, we believe that ER stress-related apoptosis plays a role in the late apoptosis of BEF cells infected with LSDV, primarily through the activation of the CCAAT/enhancer binding protein homologous protein (CHOP)-Caspase-12 signal. In addition to cell apoptosis, a further investigation showed that LSDV could also activate autophagy in BEF cells, providing additional insight into the exact causes of LSDV-induced BEF cell death. Our findings suggest that LSDV-induced BEF cell apoptosis and autophagy may provide new avenues for laboratory diagnosis of lumpy skin disease progression and exploration of BEF cell processes.

## 1. Introduction

Lumpy skin disease, caused by the lumpy skin disease virus (LSDV) belonging to the Capripoxvirus genus, is a highly contagious viral infection primarily affecting cattle. This disease leads to the formation of nodules or lumps on the skin, mucous membranes, and internal organs of infected animals. The primary mode of transmission for lumpy skin disease is through blood-feeding insects such as mosquitoes and ticks, which act as vectors. Additionally, direct contact between infected and susceptible animals can facilitate the spread of the virus. Incidences and economic destruction of this disease have been reported in various regions worldwide, including Asia, Africa, the Middle East, and parts of Europe [1,2,3,4,5]. Typical symptoms of lumpy skin disease include fever, loss of appetite, reduced milk production, and general weakness. As the disease progresses, distinct skin lesions appear, varying from small, raised bumps to large, painful nodules. These lumps often lead to secondary infections and severe discomfort for the affected animals. Due to its significant impact on livestock populations, lumpy skin disease has notable economic and trade implications. Its impact on bovine-related industries, including cattle breeding and the dairy industry, cannot be overstated [6,7,8]. Additionally, uncontrolled contamination [3,9] from LSDV raises our concern that LSDV could affect various biological products utilized in research, such as fetal bovine serum, bovine serum albumin, and bovine insulin. Hence, further exploration of the disease processes involved in lumpy skin disease is expected to provide valuable assistance to existing concerns.

LSDV belongs to the evolutionarily conserved Capripoxvirus family, sharing more than 90% homology with other members such as the goatpox virus (GTPV) and sheeppox virus (SPPV). These viruses possess a genome size of approximately 150 kilobase pairs (kbp) that encodes approximately 150 viral proteins. The high genetic similarity among these viruses determines their cross-immunogenicity [10,11], meaning protection against one virus can confer immunity against the others. Numerous studies have demonstrated the remarkable cross-protective effect of vaccines against the goatpox virus [12,13,14]. Prevention and control strategies for lumpy skin disease primarily revolve around vaccination programs, stringent biosecurity measures, and vector control. Vaccination plays a vital role in reducing disease severity and limiting its spread. Implementing quarantine measures is crucial for restricting the movement of infected animals, thus preventing further outbreaks. Moreover, examining the immune response mounted against LSDV could provide valuable insights into the development of more effective vaccines. Elucidating the specific viral proteins responsible for cross-immunogenicity between LSDV, GTPV, and SPPV may pave the way for the design of broad-spectrum vaccines capable of conferring protection against multiple Capripoxviruses. Such advancements would greatly benefit not only the bovine industry but also the broader scientific community engaged in studying these viruses. It is undeniable that immune cross-reaction based on the same genus of viruses is still an important immune measure to prevent and control the invasion of LSDV.

While vaccination plays a vital role in preventing LSDV infections, further investigation into the etiology of infected bovines is necessary to provide theoretical support and direction for the treatment of lumpy skin disease. LSDV infection in bovines results in the formation of characteristic skin lesions, manifested as numerous nodules throughout their bodies [15,16,17]. An initial investigation of LSDV in our previous study [18] demonstrated that a unique UPR strategy favors LSDV replication. However, the role of LSDV-induced endoplasmic reticulum (ER) stress in host cellular processes is still poorly understood. To further advance our understanding of LSDV, additional studies focusing on the molecular mechanisms and interactions between the virus and host cells are warranted. A deeper exploration of the UPR strategy employed by LSDV and its impact on the cellular machinery of bovine skin cells could uncover novel targets for therapeutic interventions. Additionally, investigating the signaling pathways associated with autophagy and ER-related apoptosis in the context of LSDV infection may shed light on the intricate host–virus dynamics during disease progression.

To explore ER stress-related cell death process mediated by LSDV, the following proteins are highlighted: Glucose-regulated protein 78 (GRP78), also known as Bip, is a molecular chaperone involved in protein folding and assembly. It is primarily located in the ER and plays a crucial role in regulating ER stress response and maintaining cellular homeostasis [19]. The CCAAT/enhancer binding protein homologous protein (CHOP) is a transcription factor that is induced during ER stress. It is considered a key mediator of ER stress-induced cell death [20]. Caspase-12 is an enzyme belonging to the caspase family, which plays a critical role in apoptosis, or programmed cell death. Unlike other caspases, caspase-12 is primarily localized to the ER and is specifically activated during ER stress-induced apoptosis. When Caspase-12 is cleaved, ER stress-related apoptosis is activated [21]. Microtubule-Associated Protein 1 Light Chain 3 (LC3) is a protein involved in autophagy, a cellular process that degrades and recycles damaged organelles and proteins. During autophagy, LC3 is converted from its cytosolic form (LC3-I) to a lipidated form (LC3-II), which associates with autophagosomal membranes. Monitoring LC3 levels is commonly used as a marker for autophagy activity [22].

In this study, we aimed to replicate LSDV-targeted infection in bovine skin cells, specifically using primary bovine embryonic fibroblast (BEF) cells. Through our research, we delved into autophagy driven by LSDV infection and the subsequent activation of ER-related apoptosis, which play pivotal roles in the cell death induced by LSDV. These findings provide compelling evidence that LSDV infection facilitates BEF cell autophagy and triggers the activation of ER-related apoptosis. Collectively, our results facilitate an initial characterization of the processes underlying LSDV-induced cell death in BEF cells, offering potential insights for the development of treatment strategies and laboratory diagnosis of lumpy skin disease. The devastating economic consequences and the need for effective control measures necessitate comprehensive research efforts aimed at unraveling the intricacies of LSDV infection. By understanding the molecular basis of virus–host interactions and the underlying cellular processes, we can develop targeted strategies for the prevention, treatment, and laboratory diagnosis of lumpy skin disease.

## 2. Materials and Methods

### 2.1. Cell Culture and Virus Infection

The source of BEF cells refers to our previous study [18]. To well culture the BEF cells, the following procedure was performed. First, 10% fetal bovine serum (FBS) (Gibco, Billings, MT, USA) and 1% penicillin streptomycin (Gibco, Billings, MT, USA) were supplemented into Dulbecco’s modified Eagle’s medium (DMEM) (Gibco, Billings, MT, USA) for cell culture. After that, the BEF cells were maintained in a culture environment of 37 °C and 5% CO_2_.

For the LSDV and mock infections, cells grown to approximately 70% confluence were treated with LSDV at an MOI of 1 or were mock infected with DMEM. After 1 h, the medium was changed, and the cells were maintained in 2% FBS DMEM.

The virulent LSDV strain LSDV/China/Xinjiang/2019 was provided by the Lanzhou Veterinary Research Institute, Chinese Academy of Agricultural Sciences. The strain was cultured and utilized in bio-safety level 3 (BSL-3) laboratories. When terminating experiments, a treatment that contained 2% NaOH was used for LSDV inactivation.

### 2.2. Transmission Electron Microscope (TEM) Observation

Cell samples were fixed with 2.5% glutaraldehyde, dehydrated, and embedded in resin for TEM. Ultra-thin sections were prepared using an ultramicrotome, mounted on TEM grids, and loaded into the HT7800 transmission electron microscope by Hi-tachi. The microscope operated at optimal settings to illuminate the samples with a high-energy electron beam. Interactions between electrons and internal structures generated signals captured by detectors. These signals were converted into images, revealing detailed information about cellular ultrastructure.

### 2.3. Antibodies

The primary antibodies used in this study were GAPDH (ab181603, Abcam, Cambridge, UK), GRP78 (3177S, CST, Beverly, CA, USA), LC3 (12741S, CST, Beverly, CA, USA), CHOP (5554S, CST, Beverly, CA, USA), and Caspase-12 (GB111695, Servicebio, Wuhan, China). The secondary antibodies were Goat Anti-Rabbit IgG H&L (HRP) (ab6721, Abcam, Cambridge, UK) and Goat Anti-Rabbit IgG H&L (Alexa Fluor^®^ 488) (ab150077, Abcam, Cambridge, UK).

### 2.4. Western Blotting

The complete protocol of Western blotting was conducted as follows. First, the cell samples were well prepared according to experimental requirements. Next, the cell samples were washed three times with PBS to adequately remove residual DMEM. To harvest proteins from the cell samples, cells after washing by PBS were lysed with the RIPA lysis buffer (P0013B, Beyotime, Shanghai, China) supplemented with a phenylmethylsulfonyl fluoride (PMSF) protease inhibitor (36978, Thermo, Waltham, MA, USA) and phosphatase inhibitor cocktail A (50×) (P1082, Beyotime, Shanghai, China). Lysates were transferred into new tubes, and the protein concentration was quantified via the BCA system. The lysates supplemented with the loading buffer were then boiled. The proteins were separated at 20 μg per sample by SDS-PAGE, followed by standard Western blotting. In detail, the protein sample was subjected to low-voltage electrophoresis at 80 V until it reached the interface between the concentrating gel and the separating gel. Then, the voltage was switched to 120 V until the bands ran to the bottom of the gel. After SDS-PAGE, the protein was transferred to the polyvinylidene fluoride (PVDF) membrane at a constant current of 250 mA for 55 min. To alleviate non-specific banding, the PVDF membrane was blocked for 1 h using skim milk powder. Subsequently, the PVDF membrane was cut according to the target protein and incubated overnight with the primary antibody. After the incubation of the primary antibody, the PVDF membrane was washed three times by TBST and then incubated with the secondary antibody for 1 h. After secondary incubation to target proteins, the PVDF membrane was washed three times with TBST. Finally, the proteins underwent color development.

### 2.5. Cell Apoptosis Analysis

To detect LSDV-induced cell apoptosis in BEF cells, the In Situ Cell Death Detection Kit (11684817910, Roche, Basel, Switzerland) was used to detect cell apoptosis according to the manufacturer’s instructions. According to the manufacturer’s instructions, when cells undergo apoptosis, they will exhibit bright green fluorescence under the microscope. The cells were observed under a DMI 6000B inverted fluorescence microscope (Leica, Wetzlar, Germany), and the fluorescence intensity was determined using GraphPad Prism software 6.01 (San Diego, CA, USA).

### 2.6. Cellular Indirect Immunofluorescence (IFA)

To perform indirect immunofluorescence staining, the following detailed steps were taken. First, the cell samples were gently washed three times with PBS to remove any debris or contaminants. Next, the cells were fixed using 4% paraformaldehyde (G1101, Servicebio, Wuhan, China). After fixing, the samples were washed again three times using PBS to remove excess paraformaldehyde and any remaining contaminants. To enable antibody penetration into the cells, the samples were then permeabilized with a permeabilization solution supplemented with Triton X-100. Following permeabilization, the samples were washed thrice with PBS to remove residual permeabilization agents. To minimize the non-specific binding of antibodies, a blocking solution containing 10% goat serum was used to block the cell samples. After blocking, the samples were incubated with a primary antibody specific to the target protein of interest. The primary antibody recognizes and binds to the target protein within the cell. Following primary antibody incubation, the samples underwent another round of washing three times with PBS to remove unbound primary antibodies. After that, the cell samples were labeled with Goat Anti-Rabbit IgG H&L (Alexa Fluor^®^ 488) and washed three times with PBS to remove extra secondary antibodies. Finally, cell nuclei were counterstained with DAPI (C0065, Solarbio, Beijing, China). Images were collected using a Leica SP8 confocal laser scanning device.

### 2.7. Statistical Analysis

Data were presented as the mean and standard deviation (SD). The *t*-test was performed for all of the statistical analyses using GraphPad Prism software. *p* values represent significant differences (* *p* < 0.05; ** *p* < 0.01; *** *p* < 0.001; and **** *p* < 0.0001), and ns indicates no significant difference.

## 3. Results

### 3.1. LSDV Elicits Ultrastructural Abnormalities of BEF Cells

The characteristic apparent lesions of the bovine body surface caused by bovine nodular skin infection are well known, namely, elevated skin nodules. However, there is a lack of understanding of the ultrastructural changes caused by bovine nodular skin infections. In particular, is there a compelling phenotype in the ultrastructure that matches what has been revealed about ER stress? To investigate the effects of LSDV infection on the ultrastructure of the host cell, TEM was used to observe the ultrastructure of BEF cells infected with LSDV and normal (mock infected) cells.

**Figure 1:**
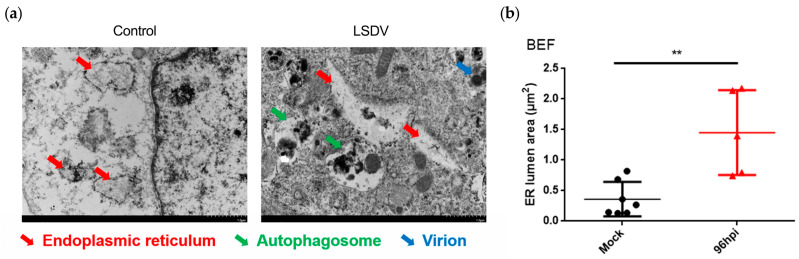
Lumpy skin disease virus (LSDV) infection causes abnormal endoplasmic reticulum (ER) ultrastructure of primary bovine embryonic fibroblast (BEF) cells. (**a**) Transmission electron microscopy (TEM) images of LSDV-infected (MOI = 1) and uninfected BEF cells at 96 hpi. The red arrows indicate the endoplasmic reticulum; the green arrows indicate autophagosomes, and the blue arrow indicates a virion); (**b**) ER lumen area of BEF cells infected or uninfected with LSDV (MOI = 1) at 96 hpi (three fields of view selected at random). The circles indicate mock cells, the triangles indicate LSDV-infected cells, and the asterisks indicate the significance.

As shown in Figure 1a, the ribosomes attached to the ER (red arrows) were located in the mock-infected BEF cells, indicating no pathological changes. Conversely, in BEF cells where virions (the blue arrow) were present, the LSDV infection resulted in an enlarged ER lumen (red arrows) in BEF cells. Further analysis suggested that the LSDV infection significantly increased the ER lumen area compared with uninfected BEF cells (Figure 1b). These phenotypes matched the occurrence of ER stress. Additionally, autophagosomes (Figure 1a, green arrows) were observed in LSDV-infected BEF cells, indicating that autophagy was occurring. Taken together, the results indicate that LSDV infection led to abnormal ultrastructural changes, as shown by the enlarged ER lumen and the formation of autophagosomes.

### 3.2. LSDV Infection Induces BEF Cell Apoptosis

Our previous studies demonstrated that LSDV induces ER stress [18], corresponding to an ER dilatation phenotype in LSDV-infected BEF cells. Hence, apoptosis driven by the ER-related death pathway was considered to possibly play a pivotal role in the infection of BEF cells by LSDV. Before exploring whether ER stress is associated with LSDV-mediated apoptosis, it is necessary to prove that LSDV infection causes BEF cell apoptosis.

**Figure 2:**
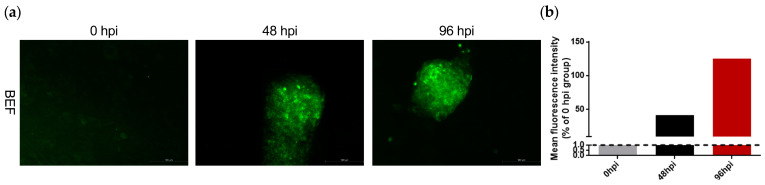
LSDV infection induces BEF cell apoptosis. (**a**) BEF cells were infected with LSDV (MOI = 1) for 0 h, 48 h, and 96 h and then stained with In Situ Cell Death Detection Kit; (**b**) the fluorescence intensity was detected to evaluate apoptosis. The dotted lines represent the 0 hpi group.

To examine this hypothesis, TUNEL staining was performed by using In Situ Cell Death Detection Kit to assess the level of apoptosis elicited by LSDV infection. As shown in Figure 2a,b, LSDV promoted BEF cell apoptosis at 48 h post-infection (hpi) presenting as highlighted green fluorescence, and this was further aggravated at 96 hpi, suggesting that LSDV infection indeed contributed to BEF cell apoptosis. These findings provide a basis for the possibility that LSDV infection may mediate the initiation of ER stress-related apoptosis programs.

### 3.3. LSDV Facilitates the Activation of ER Stress-Related Apoptosis in BEF Cells

As mentioned above, LSDV infection triggers ER stress and cell apoptosis in BEF cells, which raises the following concerns: Does endoplasmic reticulum stress induced by LSDV infection contribute to the occurrence of apoptosis during LSDV infection in BEF cells? To confirm the contribution of ER stress to BEF cell apoptosis, we further identified key markers of ER stress-related cell apoptosis in BEF cells infected with LSDV.

**Figure 3:**
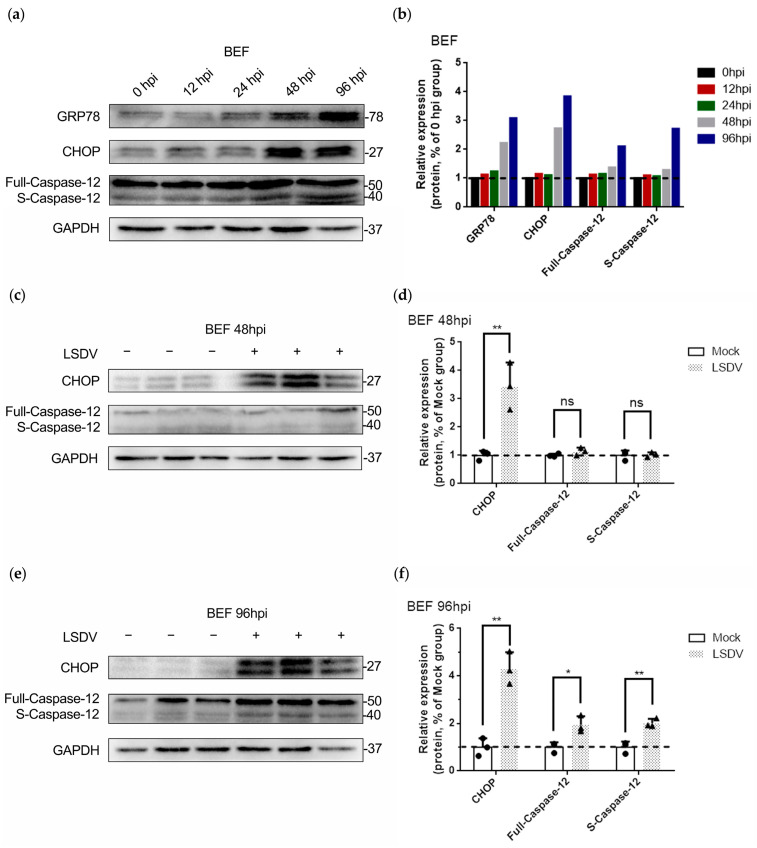
LSDV infection facilitates the activation of ER stress-related cell apoptosis in BEF cells. (**a**,**b**) BEF cells were infected with LSDV (MOI = 1) for 0 h, 12 h, 24 h, 48 h, and 96 h. Subsequently, proteins were harvested for performing Western blotting to detect ER stress-related cell apoptosis. The dotted lines represent the 0 hpi group; (**c**,**d**) BEF cells were mock infected or infected with LSDV (MOI = 1) for 48 h, and then proteins were harvested to demonstrate that ER stress-related apoptosis was not activated (*n* = 3). The dotted lines represent the mock-infected group. The circles indicate mock cells, the triangles indicate LSDV-infected cells, the asterisks indicate the significance and the ns indicates no significance; (**e**,**f**) BEF cells were mock infected or infected with LSDV (MOI = 1) for 96 h, and then proteins were harvested to demonstrate that ER stress-related apoptosis was activated (*n* = 3). The dotted lines represent the mock-infected group. The circles indicate mock cells, the triangles indicate LSDV-infected cells, and the asterisks indicate the significance.

Using gradient time infection with LSDV, we found that GRP78 expression gradually increased (Figure 3a,b), indicating that LSDV infection induced ER stress in BEF cells, especially at 48 hpi and 96 hpi. Meanwhile, the expression of the protein CHOP, a key apoptotic downstream regulator of ER stress, increased with time, especially at 48 hpi and 96 hpi. However, with LSDV infection, whether in full-length or activated sliced form, effector Caspase-12 of ER stress-related apoptosis was upregulated at 96 hpi (Figure 3a,b), indicating that ER stress may not be the only pathway of apoptosis mediated by LSDV. The expression levels of CHOP and Caspase-12 in LSDV-infected BEF cells were measured; the results suggested that significant upregulation of CHOP occurred at 48 hpi (Figure 3c,d), while significant upregulation of Caspase-12 occurred at 96 hpi (Figure 3e,f). Overall, ER stress-mediated LSDV-induced BEF cell apoptosis, but this was not the only apoptotic pathway. Intriguingly, endogenous and exogenous apoptosis may be other death pathways mediated by LSDV.

### 3.4. LSDV Infection Promotes Autophagy in BEF Cells

Above we demonstrated that LSDV infection promoted ER stress-related apoptosis. Intriguingly, apoptosis did not appear to be the only pathway toward BEF cell death during LSDV infection. We observed the presence of autophagosomes in the LSDV-infected BEF cells in the TEM images (Figure 1a, green arrows), suggesting that autophagy may also be involved in LSDV-mediated BEF cell death.

**Figure 4:**
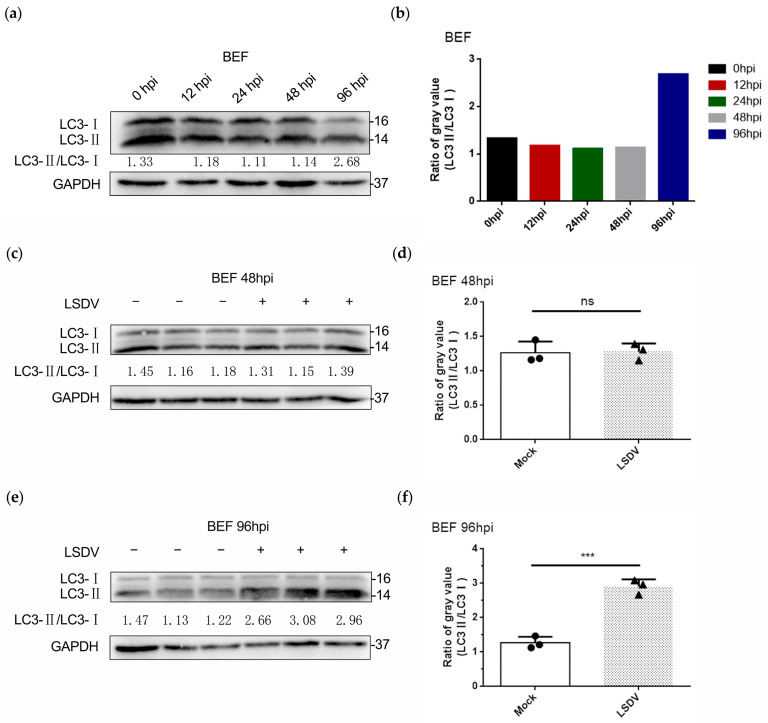
LSDV infection promotes BEF cell autophagy. (**a**,**b**) BEF cells were infected with LSDV (MOI = 1) for 0 h, 12 h, 24 h, 48 h, and 96 h. Proteins were harvested for performing Western blotting to detect the expression of the autophagy marker protein Microtubule-Associated Protein 1 Light Chain 3 (LC3) and the ratio of LC3-II/LC3-I (gray value) was analyzed; (**c**,**d**) BEF cells were mock infected or infected with LSDV (MOI = 1) for 48 h, and then proteins were harvested to demonstrate that autophagy was not activated (*n* = 3). The circles indicate mock cells, the triangles indicate LSDV-infected cells, and the ns indicates no significance; (**e**,**f**) BEF cells were mock infected or infected with LSDV (MOI = 1) for 96 h, and then proteins were harvested to demonstrate that autophagy was activated (*n* = 3). The circles indicate mock cells, the triangles indicate LSDV-infected cells, and the asterisks indicate the significance.

**Figure 5:**
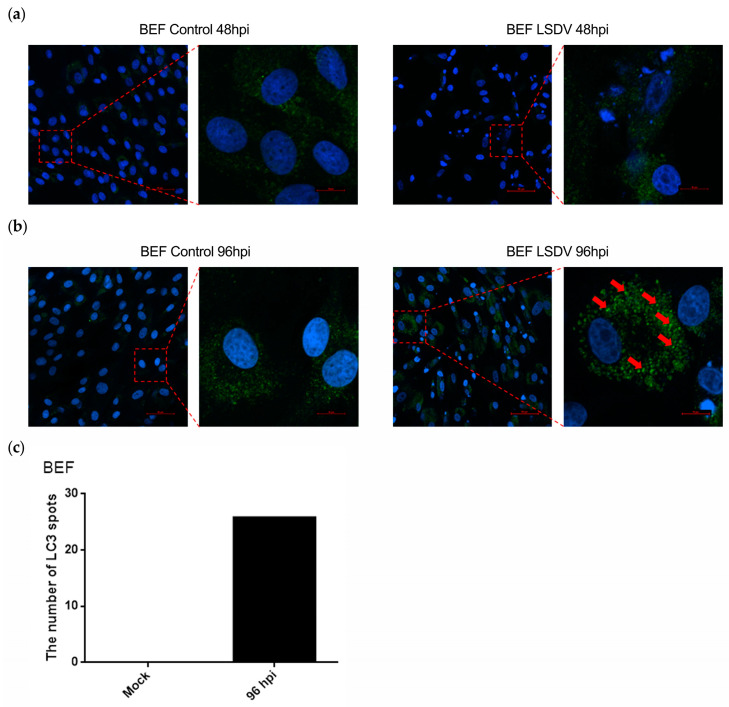
LSDV infection forms LC3 fluorescent spots. (**a**,**b**) BEF cells were mock infected or infected with LSDV (MOI = 1) for 48 h or 96 h. LC3 was fluorescently labeled to detect the formation of LC3 fluorescent spots. The red arrows indicate LC3 fluorescent spots; (**c**) the number of LC3 fluorescent spots in image (**b**) was counted.

To determine whether LSDV induced autophagy in BEF cells, the protein expression of LC3 (a marker of autophagy) was analyzed in LSDV-infected and uninfected BEF cells, and the ratio of LC3-II to LC3-I (LC3-II/LC3-I) was calculated to evaluate the level of autophagy. Notably, during the time course of infection with LSDV, we found that the ratio of LC3-II /LC3-I was unchanged at 0, 12, 24, and 48 hpi while being increased at 96 hpi (Figure 4a,b), suggesting that LSDV may induce autophagy in BEF cells. To confirm our findings, two critical time points, 48 h and 96 h, were assessed, and LC3 expression levels were further examined in infected and uninfected BEF cells. LSDV infection did not lead to autophagy (no change in the LC3-II/LC3-I ratio) in BEF cells at 48 hpi (Figure 4c,d). In contrast, the LC3-II/LC3-I ratio was significantly increased at 96 hpi compared with uninfected BEF cells (Figure 4e,f), indicating that autophagy occurred at 96 hpi. To visualize the autophagy, LC3 was fluorescently labeled, and the number of autophagy spots was counted. As described previously, at 48 hpi, no clear LC3 spots were observed in either LSDV-infected or uninfected BEF cells (Figure 5a). Remarkably, more than 20 LC3 spots were visible in BEF cells at 96 hpi, while no LC3 spots were observed in the mock-infected BEF cells (Figure 5b,c), again demonstrating autophagy in BEF cells at 96 h after LSDV infection. Taken together, the results indicate that in addition to inducing apoptosis, LSDV infection was also able to initiate autophagy in BEF cells. The determination of life processes mediated by distinct cell death programs in host cells will aid in the diagnosis and treatment of lumpy skin disease.

## 4. Discussion

Throughout history, numerous members of the poxvirus family have caused harm to human and animal health [23,24,25,26]. Well-known examples are the eradicated smallpox and the ongoing mpox epidemic. Smallpox had been with humans for centuries [27,28], killing hundreds of millions of people before being declared eradicated by the World Health Organization (WHO) in the late 20th century [29,30,31]. Unfortunately, mpox resurfaced just as COVID-19 was in decline, and the virus was declared a public health emergency of international concern by the WHO in 2022 [32,33,34], suggesting that a new poxvirus epidemic may be underway. The continued occurrence of these public health events raises concerns about the spread of the virus and requires researchers to deepen their understanding of virology. Absolutely, exploratory research based on poxvirus is in line with the current public health environment, and multidimensional research on poxvirus will aid in understanding and solving the public health risks caused by poxvirus. Hence, the analysis and investigation of infectious processes of poxvirus member lumpy skin disease virus will contribute to understanding and responding to viral disease outbreaks especially the challenges from the poxvirus members.

LSDV, a pivotal member of Capripoxvirus, can be economically devastating in its damage to cattle hides, cattle reproduction, and milk quality [35,36,37]. Ongoing research endeavors aim to develop more effective vaccines and diagnostic tools to combat lumpy skin disease. Although the prevention-based concept is widely embraced in the field of virus prevention and control, particularly for DNA viruses, it remains crucial to analyze the disease process induced by the virus itself. This analysis holds immense significance as it offers valuable insights into the diagnosis and treatment of viral diseases. Meanwhile, collaboration between veterinary authorities, farmers, and international organizations is essential to successfully manage and eradicate this disease, preserving the health and productivity of cattle populations worldwide. However, it is still unclear how LSDV interacts with host cells, especially the target BEF cells. In a prior study, we demonstrated that LSDV infection activates all three branches of the unfolded protein response in BEF cells [18]. Among these activated signals, protein kinase RNA-like ER kinase (PERK) activation is involved in mediating CHOP expression, an important regulator of ER stress-related apoptosis [38,39]. Here, TEM was performed to further visualize the abnormal ER ultrastructure in LSDV-infected BEF cells, presenting as an enlarged endoplasmic reticulum lumen compared with normal BEF cells. ER imbalance was further demonstrated. To confirm the relationship between ER stress and cell apoptosis during LSDV infection, we first demonstrated that apoptosis did occur during LSDV infection, being observed at 48 hpi and 96 hpi. However, our study showed that ER-related apoptosis was activated only at 96 hpi, suggesting that ER-related apoptosis was only involved in late LSDV-mediated apoptosis and that other processes such as endogenous (mitochondrial pathway) and exogenous (death receptor pathway) apoptosis may be involved in early LSDV-induced apoptosis. These findings reveal additional pathways by which LSDV may mediate apoptosis. In addition, we observed the presence of autophagosomes in the LSDV-infected BEF cells at 96 hpi via TEM images, suggesting a possible occurrence of autophagy during LSDV infection. The increased LC3-II/LC3-I ratio and LC3 fluorescent spot formation in LSDV-infected BEF cells implied that autophagy played an important role in the infection process of LSDV. Despite the presence of autophagy after infection with LSDV in BEF cells, no previous studies have shown that autophagy is directly related to poxvirus replication [40,41]. Therefore, autophagy can be considered to play a role in maintaining the cellular environmental balance and providing cell substrates. Autophagy represents another pathway for cell death induced by LSDV in addition to apoptosis in BEF cells.

Collectively, our study shows that the induction of apoptosis and autophagy are crucial mechanisms underlying cell death in BEF cells during LSDV infection. These findings shed light on the intricate host cell processes modulated by LSDV, leading to a better understanding of the pathogenesis of lumpy skin disease. Furthermore, our results hold significance for laboratory diagnosis of this disease. By elucidating the involvement of apoptosis and autophagy in LSDV-infected cells, we provide valuable insights for developing diagnostic assays and markers specific to lumpy skin disease. This knowledge can aid in the timely detection and accurate identification of infected animals, facilitating effective disease control and prevention strategies. Moreover, our research may even contribute to the broader poxvirus field, which aims to contribute to efforts to combat poxvirus infections and improve public health.

## Data Availability

Not applicable.
